# Determining Risk Factors for Gastric and Esophageal Cancers between 2009-2015 in East-Azarbayjan, Iran Using Parametric Survival Models

**DOI:** 10.31557/APJCP.2019.20.2.443

**Published:** 2019

**Authors:** Elaheh Zarean, Payam Amini, Mehdi Yaseri, Morteza Hajihosseini, Tara Azimi, Mahmoud Mahmoudi

**Affiliations:** 1 *Modeling in Health Research Center, School of Public Health, Department of Epidemiology and Biostatistics, Shahrekord University of Medical Sciences, Shahrekord,*; 2 *Department of Epidemiology and Reproductive Health, Reproductive Epidemiology Research Center, Royan Institute for Reproductive Biomedicine, ACECR,*; 3 *Department of Epidemiology and Biostatistics, School of Public Health, Tehran University of Medical Sciences, Tehran, Iran,*; 4 *School of Public Health, University of Alberta, Edmonton, Alberta, Canada.*

**Keywords:** Esophageal neoplasm, stomach neoplasm, survival analysis

## Abstract

**Background:**

Esophageal cancer (EC) and Gastric cancer (GC) have been identified as two of the most common cancers in the northeastern regions of Iran. The increasing rates of these types of cancers requires attention. This study aims to assess the potential risk factors for these two cancers and then determine shared risk factors between them in a population of Iranian patients using parametric survival models.

**Methods:**

This retrospective cohort study was conducted using 127 patients with EC and 184 patients with GC in East Azarbaijan, Iran who were diagnosed and registered during the years 2009-2010 in Iran’s National Cancer Control Registration Program and were followed for five years. Parametric survival models were used to find the risk factors of the patients. Akaike Information Criteria was used to identify the best parametric model in this study. Interaction analysis was used to determine shared risk factors between EC and GC.

**Results:**

The mean (±standard deviation) age of diagnoses for EC and GC were 66.92(±11.95) and 66.5(±11.5) respectively. The survival time ranges of GC patients was (0.07-70.33) and the survival time ranges were from 0.10 to 69.03 months for EC patients. Multivariable Log- logistic model showed that being married (OR=2.25, 95% CI: 1.33 - 3.81) for EC patients and Esophagectomy surgery for EC (OR: 1.62, 95% CI: 1.04 – 2.55) and GC (OR: 1.60, 95% CI: 1.02 – 2.53) had significant effects on survival. Age at the time of diagnosis, job status, and Esophagectomy surgery were statistically comparable regarding their magnitude of effect on survival of two cancers (all Ps>0.05).

**Conclusion:**

Esophagectomy surgery and being married were important risk factors in EC and GC. The log-logistic model was the most appropriate statistical approach to identify significant risk factors on survival of both cancers.

## Introduction

Gastrointestinal tract (GI) cancers including esophageal, stomach and colorectal cancer are considered to be the three most common cancers in Asia and Iran as well (Ghadimi et al., 2012; Pourhoseingholi et al., 2015). According to the International Agency for Research on Cancer (IARC), esophageal cancer (EC) is responsible for approximately 386,000 deaths per year and is considered to be the sixth most common cause of cancer worldwide. , Based on the analysis of a series of cases referred to clinics and the universities in Shiraz and Tehran, Northeastern of Iran has one of the highest rates of EC. These analyses revealed that approximately 3-4% of all registered cancers were esophageal cancer (Sadjadi et al., 2005). 

Gastric cancer (GC) is one of the most common cancers of the upper gastrointestinal tract (GI). Previous studies have shown that GC remains the seventh most common cancer in the United States and it is still the most common cancer in northern regions of Iran (Malekzadeh et al., 2009; Hu et al., 2012). Usually, patients are referred to hospitals in their advanced stages of the disease. Although the incidence of GC has decreased in recent years, approximately 990,000 people are diagnosed with GC each year worldwide and about 738,000 of them die due to GC (Karimi et al., 2014). According to the reports from the Iranian Ministry of Health and recent related studies, GI cancers such as EC and GC are the most common cancers in the East-Azarbaijan province (Naghavi, 2001; Somi et al., 2014; Darabi et al., 2016) but few studies have been conducted on the occurrence of GI cancers, their survival rates, and their related risk factors. Also, since the incidence of GI cancers in East-Azarbayjan Province is significantly high (Somi et al., 2014), identifying the potential risk factor of these fatal diseases with appropriate tools is necessary.

Due to the increasing use of survival analysis including semi-parametric and parametric multivariable survival models in medical studies, especially in cancer research, the need for efficient models with more flexibility is necessary. Despite the popularity of the semi-parametric models like the Cox Proportional Hazard model, parametric approaches can be better alternatives in some circumstances (Ghadimi et al., 2011). Although one of the most critical assumptions of the Cox Proportional Hazard Model is holding proportionality hazards (PH) assumption, in several clinical setting this underlying assumption does not hold. In such situations, accelerated failure time parametric survival techniques can be used to model risk factors for rare diseases (Kleinbaum and Klein, 2012; Cox, 2018). 

This study aimed to assess the potential risk factors of patients with EC and GC in the East Azarbaijan province of Iran using the best parametric survival model. Also, by using proper statistical methods, the shared risk factors between the two cancers were then determined for the first time in a sample of Iranian patients.

## Materials and Methods

This retrospective cohort study utilized information on patients with gastrointestinal cancers that were registered during the years 2009-2010 in Iran’s National Cancer Control Registration Program. In this national program, all pathology centers, health centers, and hospitals in provinces are obligated to report their data to the Cancer Office of Disease Control and Prevention. The data sets of this study were collected from 127 cases of patient with EC and 184 cases of patient with GC who lived in the cities of East-Azarbaijan Province. The patients that were referred to health centers and hospitals in this province were followed up for five years until 2015 and their information was extracted from their records. The patients were contacted via phone to gather information about their health and survival. The beginning of the study was assumed as the date of the pathologic diagnosis of cancer. The study outcome was considered death due to EC or GC cancer. Survival time was calculated using the difference between the dates of death and the first report of their cancer pathology. Patients who survived by the end of the study were considered as right censored.

The Two types of cancer sites included in this study were defined according to the International Classification of Diseases, 10th revision. 184 GC patients were defined by code C16 and 127 EC patients by code C15. In order to assess the potential risk factors of EC and GC, patients with prior cancers were excluded from this study. Also, there is no loss to follow-up in this study. In addition, the current study data is extracted from a MSc thesis which was checked and approved by the Ethics Committee of the TUMS (IR.TUMS.DDRI.REC.1396.4148).

The current study included three types of information: demographic, biological and socioeconomic data. The demographic variables were the age at the time of diagnosis, gender, educational status, marital status, and job status. The biological variables were non-communicable disease (NCD) affected status, Esophagectomy surgery, chemotherapy, and radiotherapy. In addition, socioeconomic status (SES) obtained based on a checklist of wealth and social position characteristics such as household fuel consumption, residential facilities, personal family facilities, and household appliances used by the family, total monthly household income, education status, and job status. Principle component factor analysis was applied to obtain the socioeconomic status (EC: KMO=0.722, Bartlett’s Sphericity test p-value<0.001; GC: KMO=0.788, Bartlett’s Sphericity test p-value<0.001). The extracted score was categorized by the median to low and high level. 

*Statistical Analysis*

Descriptive characteristics of the patients are shown as mean (± standard deviation) and frequency (percentage) for continuous and categorical variables respectively. Log-rank test was performed to assess the difference in the distribution among the levels of variables. The Cox Proportional Hazard model was performed for both EC and GC cancers, and also PH assumption was checked to take advantage of using this model in the current study. Akaike Information Criteria (AIC) was utilized to compare the performance of parametric survival models (EC AICs’ models: Log-logistic=337.94, Exponential= 343.90, Weibull=343.82; GC AICs’ models: Log-logistic=577.77, Exponential= 621.44, Weibull=601.01).

**Table 1:** The Esophageal Cancer Patients’ Characteristics and the Results of the Log-Rank Test

Variable	n (%)	Death	Log-Rank Test			Univariate log-logistic model	
		113 (89%)	Mean Survival Time	SE*	p-value	OR**	95% CI***
Age at time of diagnosis					0.384		
≥50	113 (89)	101 (89.4)	16.52	1.89		0.64	0.30-1.34
<50	14 (11)	12 (85.7)	19.71	5.02			
Gender					<0.001		
Male	70 (55.1)	68 (97.1)	10.4	1.29		0.45	0.28-0.73
Female	57 (44.9)	45 (78.9)	24.45	3.27			
Education					0.989		
Illiterate	98 (77.2)	87 (88.8)	17.14	2.08		1.16	0.66-2.04
Literate	29 (22.8)	26 (89.7)	16.21	3.43			
Marital Status					0.524		
Married	86 (67.7)	35 (85.4)	16.96	1.98		1.51	0.89-2.56
Unmarried	41 (32.3)	78 (90.7)	16.56	3.55			
Job Status					0.004		
Unemployed	79 (62.2)	67 (84.8)	20.61	2.52		1.81	1.12-2.94
Employed	48 (37.8)	46 (95.8)	10.7	1.87			
Smoking Habit					0.554		
Yes	42 (33.1)	40 (95.2)	14.39	2.21		0.93	0.56-1.54
No	85 (66.9)	73 (85.9)	18.07	2.4			
NCD affected Status					0.503		
No	103 (81.1)	93 (90.3)	16.3	1.92		0.9	0.47-1.70
Yes	24 (18.9)	20 (83.3)	18.51	4.15			
Esophagectomy surgery					<0.001		
Yes	72 (56.7)	59 (81.9)	22.48	2.8		2.08	1.30-3.32
No	55 (43.3)	54 (98.2)	9.49	1.28			
Chemotherapy					0.528		
Yes	55 (43.3)	51 (92.7)	16.8	2.1		1.01	0.99-1.03
No	72 (56.7)	62 (86.1)	16.52	2.62			
Radiotherapy					0.263		
Yes	43 (33.9)	39 (90.7)	18.08	2.6		1.48	0.92-2.41
No	84 (66.1)	74 (88.1)	16.22	2.31			
SES					0.008		
High level	66 (52)	62 (93.9)	21.57	2.9		1.82	1.12-2.95
Low level	61 (48)	51 (83.6)	12.55	1.97			

*SE, Standard Error;**OR, Odds Ratio; 95%CI, 95% Confidence Interval

**Figure1:**
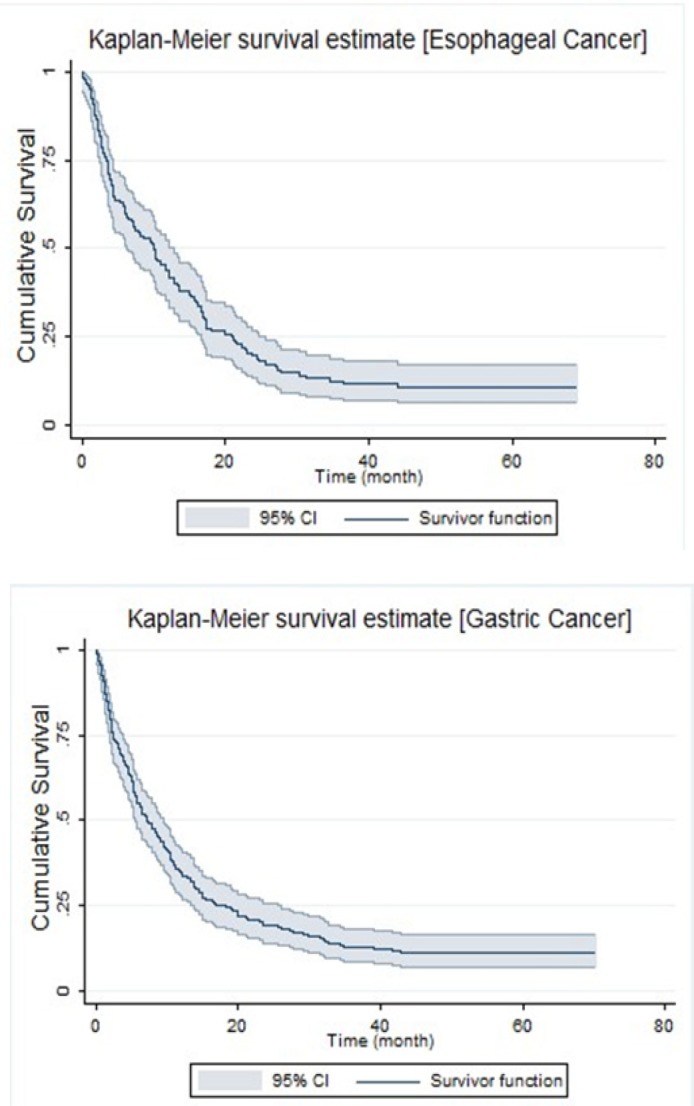
Kaplan-Meier Survival Estimate of Patients with Esophageal Cancer (Upper) and Gastric Cancer (Lower)

**Table 2:** The Gastric Cancer Patients’ Characteristics and the Results of the Log-Rank Test

Variable	n (%)	Death	Log-Rank Test			Univariate log-logistic model	
		113 (89%)	Mean Survival Time	SE*	p-value	OR**	95% CI**
Age at time of diagnosis					0.275		
≥50	166 (90.2)	147 (88.6)	15.7	1.65		0.58	0.27-1.24
<50	18 ( 9.8)	16 (88.9)	20.78	4.78			
Gender					0.303		
Male	124 (67.4)	113 (91.1)	14.8	1.75		0.88	0.55-1.42
Female	60 (32.6)	50 (83.3)	17.68	2.76			
Education					0.334		
Illiterate	112 (60.9)	100 (89.3)	15.13	1.96		0.78	0.49-1.22
Literate	72 (39.1)	63 (87.5)	17.86	2.58			
Marital Status					0.208		
Married	149 (81)	131 (87.9)	16.96	1.77		1.65	0.93-2.90
Unmarried	35 (19)	32 (91.4)	11.72	2.64			
Job Status					0.517		
Unemployed	116 (63)	100 (86.2)	17.17	2.11		1.11	0.70-1.74
Employed	68 (37)	63 (92.6)	14.38	2.16			
Smoking Habit					0.714		
Yes	62 (33.7)	56 (90.3)	14.81	2.44		0.98	0.61-1.55
No	122 (66.3)	107 (87.7)	16.44	1.97			
NCD affected Status					0.183		
No	151 (82.1)	136 (90.1)	15.14	1.65		0.7	0.40-1.27
Yes	33 (17.9)	27 (81.8)	19.59	3.78			
Esophagectomy surgery					0.001		
Yes	107 (58.2)	89 (83.2)	20.34	2.34		1.92	1.24-2.97
No	77 (41.8)	74 (96.1)	10.5	1.66			
Chemotherapy					0.027		
Yes	78 (42.4)	69 (88.5)	19.57	2.43		1.85	1.21-2.86
No	106 (57.6)	94 (88.7)	16.8	2.62			
Radiotherapy					0.16		
Yes	26 (14.1)	23 (88.5)	19.18	3.49		1.98	0.89-3.58
No	158 (85.9)	140 (88.6)	15.57	1.7			
SES					0.126		
High level	92 (50)	78 (84.4)	18.67	2.44		1.36	0.88-2.10
Low level	92 (50)	85 (92.4)	17.74	1.93			

**Table 3:** Multivariable Log-Logistic Model in Patients with Esophageal and Gastric Cancers

Variable	Esophageal Cancer		Gastric cancer		p-value***
	OR*	95% CI**	OR	95% CI	
Age at time of diagnosis (≥50)	0.9	0.43 - 1.85	0.83	0.37 - 1.88	0.135
Gender (Male)	0.33	0.16 - 0.66	0.82	0.46 - 1.47	<0.001
Education (Illiterate)	1.04	0.61 - 2.43	0.95	0.58 - 1.55	0.009
Marital status (Married)	2.25	1.33 - 3.81	1.48	0.80 - 2.73	<0.001
Job Status (Unemployed)	1.03	0.55 - 1.93	1.05	0.62 - 1.77	0.543
Smoke (Yes)	0.8	0.49 - 1.28	0.93	0.58 - 1.47	<0.001
NCD affected (No)	0.88	0.50 - 1.55	0.65	0.37 - 1.15	<0.001
Esophagectomy surgery (Yes)	1.62	1.04 - 2.55	1.6	1.02 - 2.53	0.835
Radiotherapy (Yes)	1.49	0.94 - 2.36	1.6	0.86 - 2.95	0.047
SES (High Level)	1.35	0.87 - 2.09	1.13	0.71 - 1.78	<0.001

*OR, Odds Ratio; **95% CI, 95% Confidence Interval;*** P-value, Based on analysis of the interaction between type of cancer and each risk factor, in a new model.

The univariate parametric model was used to assess the risk factors in EC and GC patients. Since the log-logistic model was recognized as the best model among the others, all the variables were entered in the multivariable Log-logistic model to find the adjusted effects of the factors on patients’ survival. Chemotherapy was removed from the multivariable model due to its high collinearity with the other predictors. The results were presented as Odds Ratio (OR) in Log-logistic models. Comparison of the estimated coefficients (beta) that resulted from the two parametric models could indicate the difference of the effect of factors on the survival of the two cancers. So, we fit another multivariable log-logistic model using a merge file of both EC and GC datasets. The type of cancer and its interactions with each of the factors is also added into this new model. These newly modified interactions assess the magnitude of the effect of various factors on the cancers. The non-significant difference between interactions estimation indicates comparable magnitude of the factor on the survival of the two cancers. All analysis performed using STATA (version 12) and the p-value<0.05 was considered statistically significant. 

## Results

The following results were found from 184 GC and 127 EC patients. The mean (± standard deviation) age of the 184 GC patients was 66.5 (± 11.5) years. The survival time ranged from 0.07 to 70.33 month and the mean and median survival time was 16.8 (95% CI: 13.6-19.9) and 8.33 (95% CI: 5.9-10.6) months respectively. One, three and five-year survival probabilities in EC and GC patients were 40.16%, 11.18%, and 11.02% for EC and 34.8%, 13% and 11.4% for GC, respectively. A total of 163 (86.6%) individuals experienced death due to GC by the end of the study. Moreover, the mean (± standard deviation) age of the 127 patients with esophageal cancer was 66.92 (± 11.95) years. The survival time ranged from 0.10 to 69.03 months and the mean and median survival time was 16.99 (95% CI: 13.46-20.52) and 10.06 (95% CI: 6.49-13.63) months respectively. A total of 113 patients (89%) experienced death due to esophageal cancer by the end of the study. The Kaplan-Meier survival estimate of patients with Esophageal and Gastric Cancers are showed in Figure 1 since PH assumption in the Cox regression model did not hold in the dataset of patients with EC (p-value=0.035) and GC (p-value= 0.044), parametric models can be selected as useful alternatives in this study. According to the results of AICs from multivariable parametric models, the Log-Logistic model had the best fitting distribution compared to other parametric models. 

The patients’ characteristics, log-rank test and univariate log-logistic model for EC and GC are shown in Tables 1 and 2 respectively. According to the log-rank results, EC survival time was significantly longer among females, unemployed cases, those who had Esophagectomy surgery, and patients with high socioeconomic status. Gastrointestinal cancers survival-time was influenced by Esophagectomy surgery and chemotherapy. Accordingly, the results of the univariate log-logistic models showed that survival in patients with EC is affected by being male (OR=0.45; 95% CI: 0.28-0.73), unemployed(OR=1.81; 95% CI: 1.12-2.94), having Esophagectomy surgery (OR=2.08; 95% CI: 1.30- 3.32) or radiotherapy (OR=1.69; 95% CI: 1.04- 2.75) and high-level SES (OR=1.82; 95% CI: 1.12-2.95). Additionally, the odds of survival in patients with GC was affected by having Esophagectomy surgery (OR=1.92; 95% CI: 1.24-2.97) and chemotherapy (OR=1.85; 95% CI: 1.21-2.86).

The results of multivariable Log- logistic model in patients with EC and GC are shown in Table 3. Based on the multivariable 95% CI for the odds ratios, married EC patients were 2.25 (95% CI: 1.33 - 3.81) times more likely to survive than unmarried patients. Patients who had Esophagectomy surgery had a 62% increase of odds of survival (OR: 1.62, 95% CI: 1.04 – 2.55). However, survival after diagnosis of GC was only affected by Esophagectomy surgery (OR: 1.60, 95% CI: 1.02 – 2.53). 

Based on the p-values in Table 3, gender, smoking habit and radiotherapy affected GC survival more than EC survival. In contrast, EC survival was more affected by education, marital status, NCD diagnosis status and SES comparing to GC. Age at the time of diagnosis, job status, and having Esophagectomy surgery showed a similar magnitude of effect on both of the cancers.

## Discussion

In this paper, we investigated the possible association between the survival of the patients with EC and GC and several of the most common prognosis factors. We assessed the impact of age at the time of diagnosis, gender, education, marital status, job status, smoking habit, NCD diagnosis status, Esophagectomy surgery, chemotherapy, radiotherapy and socioeconomic status (SES). In the current study, being male was a significant indicator of prognosis in both univariate and multivariable analysis in patients with EC. This finding supports findings from Chen et al. where being male was found to significantly reduce patients’ survival rates (Eil et al., 2014). In our study, high-level socioeconomic status had a positive influence on the survival of EC patients in the univariate Log-logistic model, which was similarly found in a study by Gammon et al., (1997). Our findings also indicated that Esophagectomy surgery had a significant effect on the survival odds of patients with EC in both univariate and multivariable Log-logistic approaches. Previous reports have suggested that Esophagectomy surgery increases the odds of survival among EC patients (Chen et al., 2013). In the current study, married patients in the EC group had significantly higher odds of death in the multivariable Log-logistic model. Previous studies have also demonstrated that a patient’s survival is affected by their marital status (Ernster et al., 1979; Aizer et al., 2013). The current study has found controversy in the results of univariate and multivariable approaches regarding radiotherapy. Using an intergroup phase III randomized clinical trial, Al-saraf et al., (1997) compared the effect of combined chemotherapy-radiotherapy versus radiotherapy only in patients with locally advanced EC. They found that the median survival of patients with combined chemotherapy-radiotherapy was higher than that of radiotherapy alone. Our study has shown that Esophagectomy surgery is an essential and outstanding prognostic indicator of GC survival in both the univariate and multivariable Log-logistic model. Improving survival followed by surgery was assessed by Hallissey et al., (1994) on a group of British GC patients with stomach cancer. They concluded that surgery is the standard treatment for GC. However, our findings are not consistent with results from the prospective randomized controlled trial study by Allum et al., (1989) where the effects of surgery with adjuvant radiotherapy and chemotherapy in patients with operable GC were evaluated. Neither forms of adjuvant therapy were associated with the survival of GC patients nor surgical treatment remained the principal treatment. 

Parametric and semi-parametric survival models have been used widely in fitting survival data. This might be due to the fact that the parametric approaches such as Log-logistic, Weibull, and Exponential models provide accurate estimates with some predetermined assumptions (Efron, 1977). The AIC scores in our study revealed that the Log-logistic model was the best-fitted model in GC and EC datasets. Findings from Ghadimi et al., (2012)’s study on patients with GC in the city of Babol, Iran was consistent with the findings in the present study. The result of our study indicated that in the multivariable log-logistic model, the odds ratio of survival among age≥50 in GC is lower than EC. Roshanaei et al., (2010) conducted a study regarding the survival of patients with GC under surgery in which gender pathologic stage, age at diagnosis and weight-loss were significantly related to the survival of the patients in the multivariable analysis (Roshanaei et al., 2010). The effect of age at diagnosis has been discussed by Zare et al. They demonstrated that the age at diagnosis has a significant effect on the survival of patients with GC who have undergone surgery (Zare et al., 2014). Moreover, Greenstein et al. found that being over 70 reduced the chance of survival for patients diagnosed with esophageal cancer (Greenstein et al., 2008). 

This study exposed that GC patients are more likely to survive after radiotherapy compared to EC patients. Moreover, SES has a greater effect on survival time among EC patients compared to patients with GC. The present study showed that females in both cancers have higher odds of survival compared to males. This might be related to their riskier lifestyles (Arnal et al., 2015). However, males with EC live shorter lives than those with GC. In another study in China, Chen et al. revealed that the rate of mortality and incidence of EC in males was higher than those of patients with GC (Chen et al., 2016). Our study showed that married people have higher odds of survival. Moreover, those with EC have a higher survival time than those with GC. Lagergren et al. assessed the impact of marital status, education, and income on the risk of esophageal and gastric cancers. They showed that patients with long marriages have lower incidence rate ratios compared to those with shorter marriages or those who were never married, remarried, or divorced. The ratios were lower among EC patients in comparison to patients with GC (Lagergren et al., 2016). 

There were some limitations on the relatively small sample size in our data. The most important limitation of the survey was the absence of clinical information including the esophageal and gastric cancer type and the stage of these cancers. Since this was a retrospective cohort study, we did not have access to the information on the exposures that patients encountered. 

We conclude that marital status and Esophagectomy surgery were potential risk factors for the survival of EC patients. Surgical techniques may be a useful method to increase the survival rate of patients with esophageal cancer. Radiotherapy is an appropriate treatment and may decrease death caused by EC. In patients who have already had GC surgery, chemotherapy and radiotherapy are alternative treatment approaches to increase the survival chances of patients with gastric cancer. We founded that the Log logistic model could be a proper approach for statistical analysis of risk factors in patients with EC and GC. 
